# Protective Efficacy in a Hamster Model of a Multivalent Vaccine for Human Visceral Leishmaniasis (MuLeVaClin) Consisting of the KMP11, LEISH-F3+, and LJL143 Antigens in Virosomes, Plus GLA-SE Adjuvant

**DOI:** 10.3390/microorganisms9112253

**Published:** 2021-10-29

**Authors:** Laura Fernández, Jose Carlos Solana, Carmen Sánchez, Mª Ángeles Jiménez, Jose M. Requena, Rhea Coler, Steven G. Reed, Jesus G. Valenzuela, Shaden Kamhawi, Fabiano Oliveira, Epifanio Fichera, Reinhard Glueck, Maria Elena Bottazzi, Gaurav Gupta, Pedro Cecilio, Begoña Pérez-Cabezas, Anabela Cordeiro-da-Silva, Luigi Gradoni, Eugenia Carrillo, Javier Moreno

**Affiliations:** 1 WHO Collaborating Centre for Leishmaniasis, Centro Nacional de Microbiología, Instituto de Salud Carlos III, 28220 Madrid, Spain; laura.fernandezfer@hotmail.es (L.F.); csanchezh@isciii.es (C.S.); ecarrillo@isciii.es (E.C.); javier.moreno@isciii.es (J.M.); 2 Facultad de Veterinaria, Departamento de Medicina y Cirugía Animal, Universidad Complutense de Madrid, 28040 Madrid, Spain; mariadji@vet.ucm.es; 3 Centro de Biología Molecular Severo Ochoa (CSIC-UAM), Universidad Autónoma de Madrid, 28049 Madrid, Spain; jmrequena@cbm.csic.es; 4 Center for Global Infectious Disease Research (CGIDR), Seattle Children’s Research Institute, Seattle, WA 98109, USA; Rhea.Coler@seattlechildrens.org; 5 HDT Bio Corp, Seattle, WA 98102, USA; steven.reed@idri.org; 6 Vector Molecular Biology Section, Laboratory of Malaria and Vector Research, NIAID, NIH, Rockville, MD 20852, USA; jesus.valenzuela@nih.gov (J.G.V.); skamhawi@niaid.nih.gov (S.K.); loliveira@niaid.nih.gov (F.O.); 7 Etna Biotech S.R.L, 95121 Catania, Italy; epifanio.fichera@etnabiotech.it (E.F.); recafra@gmail.com (R.G.); drgaurav123@gmail.com (G.G.); 8 Department of Pediatrics, Texas Children’s Center for Vaccine Development, National School of Tropical Medicine, Baylor College of Medicine, Houston, TX 77030, USA; bottazzi@bcm.edu; 9 Parasite Disease Group, Instituto de Investigação e Inovação em Saúde (i3S), Universidade do Porto, 4200-135 Porto, Portugal; pedro_cecilio_28@msn.com (P.C.); bperezcabezas@gmail.com (B.P.-C.); cordeiro@ibmc.up.pt (A.C.-d.-S.); 10 IBMC-Instituto de Biologia Celular E Molecular, Universidade do Porto, 4150-180 Porto, Portugal; 11 Faculdade de Farmácia da, Universidade do Porto, 4099-002 Porto, Portugal; 12 Unit of Vector-Borne Diseases, Istituto Superiore di Sanità, 00161 Rome, Italy; luigi.gradoni@iss.it

**Keywords:** leishmaniasis, hamster, vaccine, virosomes, KMP11, LEISH-F3, LJL143, GLA-SE

## Abstract

Visceral leishmaniasis (VL) is the most severe clinical form of leishmaniasis, fatal if untreated. Vaccination is the most cost-effective approach to disease control; however, to date, no vaccines against human VL have been made available. This work examines the efficacy of a novel vaccine consisting of the *Leishmania* membrane protein KMP11, LEISH-F3+ (a recombinant fusion protein, composed of epitopes of the parasite proteins nucleoside hydrolase, sterol-24-c-methyltransferase, and cysteine protease B), and the sand fly salivary protein LJL143, in two dose ratios. The inclusion of the TLR4 agonist GLA-SE as an adjuvant, and the use of virosomes (VS) as a delivery system, are also examined. In a hamster model of VL, the vaccine elicited antigen-specific immune responses prior to infection with *Leishmania infantum*. Of note, the responses were greater when higher doses of KMP11 and LEISH-F3+ proteins were administered along with the GLA-SE adjuvant and/or when delivered within VS. Remarkably, hamsters immunized with the complete combination (i.e., all antigens in VS + GLA-SE) showed significantly lower parasite burdens in the spleen compared to those in control animals. This protection was underpinned by a more intense, specific humoral response against the KMP11, LEISH-F3+, and LJL143 antigens in vaccinated animals, but a significantly less intense antibody response to the pool of soluble *Leishmania* antigens (SLA). Overall, these results indicate that this innovative vaccine formulation confers protection against *L. infantum* infection, supporting the advancement of the vaccine formulation into process development and manufacturing and the conduction of toxicity studies towards future phase I human clinical trials.

## 1. Introduction

Leishmaniasis is responsible for one of the highest rates of disability-adjusted life years (DALYs) caused by any neglected tropical disease [1]. It affects 12 million people worldwide, and approximately 350 million people are at risk [2]. Visceral leishmaniasis (VL), caused by *Leishmania infantum* and *Leishmania donovani*, is the most severe form of the disease, leading to death if left untreated [3].

Control of the leishmaniases depends on the identification of clinical cases and reservoir hosts and their treatment. However, most anti-*Leishmania* drugs available can cause adverse side effects (including toxicity), are difficult to administer, and face the problem of emerging resistance [4,5]. Since cured patients are resistant to re-infection, vaccination is thought to be the most cost-effective way of gaining control over this disease [6]. Certainly, vaccines against canine leishmaniasis have been developed; these are based either on parasite protein extracts (CaniLeish) [7]) or recombinant proteins (Leish-Tec [8] and LetiFend [9]) and have all been marketed at some point in Brazil or Europe [10,11]. However, there is still no effective vaccine approved for human use [12]. The present work aimed to assess the protective potential of a new vaccine formulation based on two parasite antigenic elements—the membrane protein KMP11 and the fusion protein LEISH-F3+—plus the salivary protein LJL143 from the sand fly *Lutzomyia longipalpis*, vehiculated in virosomes and adjuvanted by a strong TLR-4 agonist, against *L. infantum* infection.

KMP11 is a well-known antigen that has already been used in pre-clinical experiments in the form of a DNA vaccine [13] and chimeric vaccines [14,15], one of which—against VL and post-kala-azar dermal leishmaniasis—is now being tested in the context of phase I clinical trials [16]. Although the efficacy of vaccines based on KMP-11 depends on the nature of the antigen, most of the studies performed as DNA vaccines were successful against *Leishmania* [14,15]. LEISH-F3 is a recombinant fusion protein based on the parasite proteins nucleoside hydrolase (NH) and sterol-24-c-methyltransferase (SMT). It conferred protection against *Leishmania* infection in rodent models and elicited a Th1-like immune response in humans, as per the results of a phase I clinical trial [17]. In an attempt to increase its antigenic diversity, a new version of this protein was recently developed that includes a third component derived from *Leishmania* cysteine protease B (CPB). This new protein, LEISH-F3+, shows immunogenic and prophylactic properties that can be superior to those of LEISH-F3 in mouse and hamster models [18,19].

Sand fly saliva is composed of pharmacologically active components (sialogenins) with anti-hemostatic, anti-inflammatory, and immunomodulatory properties that play important roles in *Leishmania* infection by impairing anti-parasitic immune responses [20,21]. This highlights the need to include vector elements in vaccine strategies [20]. In fact, some sand fly salivary proteins conferred protection against *Leishmania* infection—alone [22,23,24], or in combination with other vaccine elements as a way to generate wider and stronger immune responses [25]. For example, the *Lu. longipalpis* salivary protein LJL143 induces humoral responses and a Th1-cytokine profile in mice [26] and dogs [23,27].

Glucopyranolsyl lipid A, when formulated in a stable oil-in-water nanoemulsion (GLA-SE), functions as a TLR4 agonist that induces a Th1-response. Importantly, it has been previously used in human clinical trials as an adjuvant to enhance the immune response against *Leishmania* and other pathogens [17,28,29,30].

Virosomes (VS) are one of the few adjuvant systems approved by regulatory authorities for human use which have carrier capabilities. The technology platform is robust enough to allow for the efficient loading of a variety of antigens such as proteins, peptides, carbohydrates, and nucleic acids; importantly, the production level can be easily scaled up. The above characteristics, together with excellent safety records, make virosomes suitable delivery systems for a vaccine against VL [31,32,33]. Their main targets are dendritic cells; they enhance antigen uptake and promote cytotoxic T cell-mediated immune responses [34,35].

The first step in the development of any vaccine involves preclinical animal testing in a variety of different species and models. Animal models such as rodents (mice, hamsters), dogs, and primates (non-human) are used to study visceral leishmaniasis. BALB/c mice are the main immunological model of infection used for the pre-clinical evaluation of drugs and vaccines [36,37,38,39]. Previous studies undertaken by our consortium (the EU-FP7 MuLeVaClin consortium) have shown that different combinations of the antigens KMP11, LEISH-F3+, and LJL143, in conjunction with GLA-SE and VS, induce a Th1-type immune response in BALB/c mice, which might be protective against subsequent *Leishmania* infection [26]. The latter study also showed LJL143 to be the most immunogenic element tested, inducing a strong cellular response elicited via IFN-γ and IL-10.

On the contrary, hamsters are an excellent model for studying the pathogenesis of VL; in fact, in contrast to the self-resolving mouse model, the hamster model of visceral leishmaniasis parallels human disease [40]. Therefore, the infection of hamsters with *L. infantum* and *L. donovani* is considered the best experimental model available to assess the efficacy of drugs and vaccines against VL [40]. However, good results in animal models do not guarantee success when applying the same formulation to humans.

The present work aimed to test the efficacy of this vaccine formulation in hamsters after intracardiac *L. infantum* infection, a model suitable for the study of VL and its clinical progression [41].

## 2. Materials and Methods

### 2.1. Ethics Statement

All procedures described here were approved by the Committee on Ethics and Animal Welfare of the *Instituto de Salud Carlos III* (CBA 06_2014) and performed according to the Spanish legislation on the protection of animals for experimentation and other scientific purposes (Royal Decree 53/2013, law 32/2007), which adheres to the European Directive 86/609/EEC.

### 2.2. Experimental Animals, Parasites, Antigens, and Adjuvant

Animals and parasites. The VL model animals used in this work were golden hamsters (*Mesocricetus auratus*) (Janvier, Le Genest-Saint-Isle, France); the parasite species used was *L. infantum* (MCAN/ES/98/LLM-724, JPC strain).

Soluble *Leishmania* antigen (SLA). This antigen pool was prepared from *L. infantum* promastigotes using a freeze–thaw procedure [42]. Briefly, promastigotes were washed in PBS and centrifuged at 1000× *g* for 20 min at 4 °C. They were then suspended in 50 mM of Tris-HCl and 5 mM of EDTA, pH 7, and subjected to freeze–thaw cycles, sonicated, and centrifuged again at 27,000× *g* for 20 min at 4 °C. The supernatant containing the soluble proteins was centrifuged at 100,000× *g* for 4 h at 4 °C before being aliquoted under sterile conditions and stored at −80 °C. The protein concentration of the extract was determined using the Pierce BCA Protein Assay Kit (Thermo Fisher Scientific, Waltham, MA, USA) following the manufacturer’s instructions. SLA is not part of the vaccine’s composition; it was only used in immunological assays.

KMP11. The *L. infantum* gene coding for KMP11 (LinJ.35.2260) was cloned into the vector pET-28b for expression in *E. coli* BL21, as previously described [26]. The gene was amplified from *Leishmania* genomic DNA using the oligonucleotides 5′-CCATGGCCACCACGTACGAGG and 5′-GGATCCTTACTTGGACGGGTACTGCG, avoiding the addition of any tag for protein purification purposes in order to fulfill the requirements of good manufacturing practices. The protein was purified by ammonium sulfate precipitation (at least 80% ammonium sulfate saturation) and by anion-exchange chromatography. KMP11 was then eluted in 10 mM of Tris-HCl, pH = 8.5, and 150 mM of NaCl and passed through a polymyxin-B agarose matrix (Sigma-Aldrich, St. Louis, MO, USA) for endotoxin removal. The purity was >95%, as determined by SDS-PAGE and Coomassie staining.

LEISH-F3+. The chimeric protein LEISH-F3+ was originated by the fusion of the *Leishmania* CPB antigen and LEISH-F3 [18]. The production and purification processes were similar to those previously described [17].

LJL143. The sand fly salivary protein was produced using a mammalian expression system, as explained elsewhere [23]. Briefly, DNA coding for LJL143 was subcloned into the *Pichia pastoris* secretory expression vector pPICZαA (Invitrogen, Carlsbad, CA, USA) between the EcoRI/XbaI restriction sites. The correct insertion sequence was confirmed by double-stranded sequencing using the vector flanking primers α-factor and 3′AOX-1 before electroporation into *Pichia pastoris* X-33. The expression of LJL143 was induced with 0.5% methanol at 30 °C for 72 h. The purity of the expressed protein was confirmed by Coomassie staining (NuPAGE Bis-Tris gel; Invitrogen, Waltham, MA, USA).

Adjuvant. The synthetic TLR-4 agonist glucopyranosyl lipid A in stable emulsion (GLA-SE) was produced and provided by the Infectious Disease Research Institute (IDRI; Seattle, WA, USA) [43].

### 2.3. Virosome-Based Antigens

Virosomes containing each of the antigens individually were prepared, as previously described [26]. Briefly, a mixture of 1 mg of inactivated influenza virus A/H1N1/California and 32 mg of phosphatidylcholine (Lipoid Ag, Steinhausen, Switzerland) was centrifuged at 100,000× *g* for 30 min, and the supernatant, containing haemagglutinin and neuraminidase, was recovered. These influenza antigens were then used to produce VS that included the vaccine antigens (individually) by detergent removal. The VS were then sterile-filtered and their size distribution was determined using a Zetasizer Nano instrument (Malvern Instruments, Malvern, UK). The parasite-derived and/or virus-derived protein contents of the VS were checked by SDS-PAGE Coomassie staining.

### 2.4. Immunization and Infection

Hamsters were distributed into six groups (9 animals per group) and immunized intramuscularly with 100 µL of the different vaccine formulations (see below) three times at four-week intervals. The antigens were administered to animals in different groups at different concentrations (except for LJL143, which was limited to 1 µg to prevent immunodominance), combined or not with the adjuvant GLA-SE. Briefly, the animals in groups PA 1+1+1 and PA 5+5+1 (see legend to Table 1) were immunized with 1 or 5 µg of KMP11 and LEISH-F3+ proteins, respectively, plus 1 µg of LJL143 in combination with 1 µg of the GLA-SE adjuvant. The groups VPA 1+1+1 and VPA 5+5+1 received the same doses as the previous groups, individually within VS (i.e., VS-KMP11, VS-LEISH-F3+, and VS-LJL143 associations). Animals in group P 5+5+1 were immunized with the antigen mix (5 µg of KMP11 and LEISH-F3+ and 1 µg of LJL143) without an adjuvant or VS. The control group animals received only PBS (Table 1).

Four weeks after administration of the last dose, the animals were infected intracardially with 2 × 10^7^ *L. infantum* promastigotes in the stationary phase [44,45]. One hamster of the VPA 5+5+1 group died after infection; this group, therefore, had eight animals instead of nine. Twelve weeks after infection, all animals were anesthetized with 2% isoflurane (Verflurano; Virbac, Carros, France) and euthanized by cardiac puncture.

### 2.5. Whole Blood Collection

One day before infection, each animal was anesthetized with isoflurane 2% and a small sample of was blood collected from the carotid vein in an EDTA-Ca^2+^ tube (Microvette; Sarstedt, Numbrecht, Germany) [46] to obtain plasma. During euthanasia, approximately 4 mL of blood were extracted from each hamster and collected in lithium heparin tubes (Sarstedt) to obtain plasma and to isolate peripheral blood mononuclear cells (PBMCs).

### 2.6. Cell Proliferation Assay

PBMCs were isolated from blood obtained during euthanasia using a Ficoll–Hypaque density gradient (Lymphocyte Isolation Solution; Rafer, UK) as previously described [44]. Then, 1 × 10^5^ PBMCs/well were plated into 96-well microplates in an RMPI 160 medium supplemented with 2 mM of L-glutamine, 25 mM of Hepes, 100 U/mL of penicillin, 100 µg/mL of streptomycin (all from Lonza, Basel, Switzerland), and 10% heat-inactivated fetal bovine serum (FBS; Sigma-Aldrich, St. Louis, MO, USA). Cells were stimulated for 5 days (37 °C, 5% CO_2_) with 10 µg/mL of the individual vaccine antigens (KMP11, LEISH-F3+, or LJL143) or with SLA. Concanavalin A (5 µg/mL) (Sigma-Aldrich, St. Louis, MO, USA) and supplemented RPMI were used as positive and negative controls of the assay, respectively. BrdU was added to each well during the last 24 h of culture, to examine lymphocyte proliferation using the ELISA GE Healthcare Cell Proliferation Kit (GE Healthcare Life Sciences, Marlborough, MA, USA).

### 2.7. Humoral Response Analysis

Plasma was obtained after blood centrifugation (500× *g* for 10 min at 4 °C) and stored at −20 °C until use. The presence and reactivity of IgG antibodies against SLA, KMP11, LEISH-F3+, and LJL143 were determined by ELISA. Briefly, Nunc Maxisorp 96-microtiter well plates (Thermo Fisher Scientific, Waltham, MA, USA) were coated overnight at 4 °C with 1 µg/well of SLA, KMP11, LEISH-F3+, or LJL143 in carbonate buffer (Na_2_CO_3_ 15 mM, NaHCO_3_ 39 mM, pH 9). The plates were then washed three times with PBS 0.01% Tween 20 (Sigma-Aldrich, St. Louis, MO, USA) and coated with 1% bovine serum albumin (BSA) in PBS (Sigma-Aldrich, St. Louis, MO, USA) (blocking solution) for 1 h at 37 °C. Plasma samples were then added—diluted 1/100 in blocking solution—for 1 h at 37 °C. For IgG detection, the wells were washed and incubated for 1 h at 37 °C with IgG α-hamster peroxidase-conjugated secondary antibodies (Abd Serotec, Oxford, UK) diluted 1/5000 (SLA and LEISH-F3+) or 1/1000 (KMP11 and LJL143). Finally, the plates were washed and incubated with o-phenylenediamine for 15 min. The colorimetric reaction was stopped using H_2_SO_4_ 1M and the optical density was measured at 490 nm in a spectrophotometer (Thermo Fisher Scientific) to determine reactivity against each antigen.

### 2.8. Parasite Load Quantification by PCR

Following necropsy, liver and spleen samples of each animal were homogenized in supplemented RPMI (see above) using a 40 µM Falcon Cell Strainer (Thermo Fisher Scientific, Waltham, MA, USA). To obtain DNA, around 3 × 10^6^ cells were lysed with 400 μL of NET10 buffer (NaCl 10 mM, EDTA 10 mM, Tris-HCl 10 mM, pH 8.0), 40 μL of SDS 10% and 0.1 mg/mL of proteinase K (20 mg/mL) (Sigma-Aldrich, St. Louis, MO, USA) for 1 h at 70 °C. The DNA was then extracted with phenol:chloroform:isoamyl alcohol (25:24:1) (Sigma-Aldrich) [47] and dissolved in 100 μL of nuclease-free water. The DNA concentration was measured using a NanoDrop^®^ 1000 spectrophotometer (Thermo Fisher Scientific).

The *Leishmania* DNA was quantified by quantitative real-time PCR (qPCR) in 4 µL of DNA from the different samples, using a LightCycler 2.0 thermocycler (Roche, Basel, Switzerland), 1000 nM of primer R223, 500 nM of primer R333, 2.5 mM of MgCl_2,_ and LightCycler FastStart DNA Master SYBR Green I (Roche) [44]. The parasite numbers in each sample were obtained using a standard curve [48]. The final results are expressed as relative units (RU) (i.e., with respect to the mean value of the control group) [49]. In addition, the mean RU of each group was calculated for comparison with that of the control group (considered as 1), and the difference is expressed as the percentage reduction or increase in parasite burden.

### 2.9. Histopathology

Liver samples were collected after euthanasia, fixed in 10% buffered formalin, trimmed transversely along the main lobule, embedded in paraffin blocks, and processed and stained with hematoxylin and eosin following standard laboratory procedures. Two step sections were examined under light microscopy by a trained pathologist (blind to the study). The total number of granulomas in each section was counted and classified according to their maturation status.

The efficiency of granuloma maturation and organization has been associated with the host capacity to resist infection in human, murine, and canine models [14,50,51,52]. Moreover, the effectiveness of protozoan vaccines has been related to the capacity to induce sterile granulomas [14]. Thus, granulomas were classified and counted as immature (developing granulomas), organized (distinct granulomas) or sterile (distinct, organized granulomas with central Kupffer cells and macrophages, surrounded by mixed inflammatory cells with a predominance of lymphocytes), according to previously reported classifications [14,51].

### 2.10. Statistical Analysis

The normality of the data was examined using the D’Agostino–Pearson test and the experimental groups were consequently compared using the Mann–Whitney U test. Significance was set at *p* < 0.05. All calculations were made using GraphPad Prism v.8.01 software (GraphPad Software Inc., San Diego, CA, USA).

Histopathology results were analyzed using a non-parametric ANOVA Kruskal–Wallis test with SPSS software (IBM Inc. SPSS Inc. statistics).

## 3. Results

### 3.1. Antigenicity of the Different Vaccine Formulations

**Figure 1:**
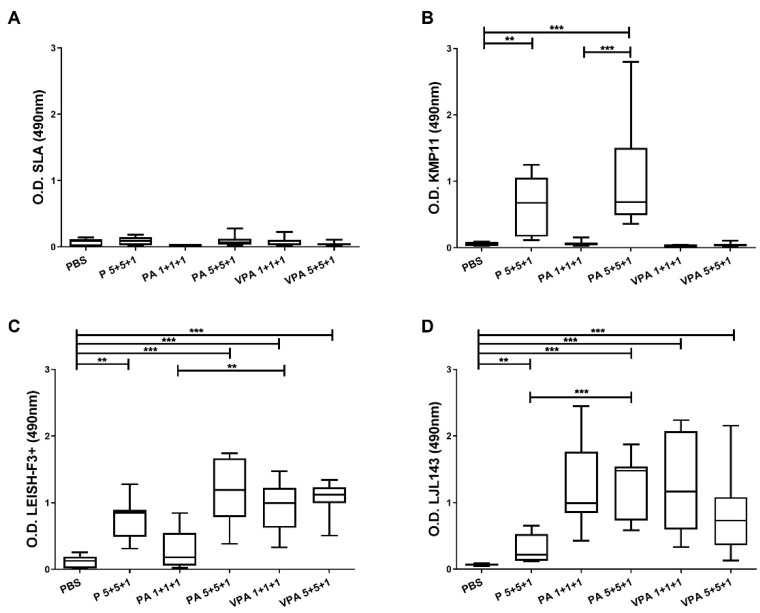
Humoral responses in immunized animals. Four weeks after the last immunization, IgG reactivity against *L. infantum* SLA (**A**), KMP11 (**B**), LEISH-F3+ (**C**), and LJL143 (**D**) was determined by ELISA in plasma from immunized hamsters (*n* = 9 per group). Results are shown as whisker (min to max) plots. Statistical differences between groups are indicated (Mann–Whitney U test): ** *p* < 0.01; *** *p* < 0.001.

Hamsters (9 animals per group) were immunized three times intramuscularly at four-week intervals with PBS or different permutations of the vaccine components. The P 5+5+1 group received the non-formulated and non-adjuvanted proteins KMP11 (5 µg), LEISH-F3+ (5 µg), and LJL143 (1 µg). The PA groups received the proteins and the adjuvant GLA-SE in two different combinations: 1 µg of each protein (PA 1+1+1) or 1 µg of LJL143 and 5 µg of KMP11 and LEISH-F3+ (PA 5+5+1). The VPA groups received the GLA-SE-adjuvanted antigens formulated in virosomes using the same dosages as above (VPA 1+1+1 and VPA 5+5+1). Four weeks after the third and final immunization—just before performing infection—blood samples were obtained to evaluate vaccine antigenicity. A very low IgG response to SLA was detected; of note, no differences were found between the vaccinated and control groups (Figure 1A). However, the immunized animals showed a specific antibody response against the different vaccine antigens. Anti-KMP11 antibodies were found in animals immunized with 5 µg of KMP11 and LEISH-F3+ proteins (P 5+5+1), but not in animals immunized with 1 µg (P 1+1+1) or those in the VPA 1+1+1, VPA 5+5+1 groups (Figure 1B). Interestingly, the presence of the adjuvant GLA-SE in the PA 5+5+1 group increased serum reactivity against KMP11 compared to that recorded for the group immunized with proteins alone (*p* = 0.0133). Humoral response against the chimeric protein LEISH-F3+ was present in nearly all the immunized animals—especially in the PA 5+5+1 and both VPA groups (Figure 1C). The exception was the group PA 1+1+1; the animals showed responses similar to those seen in the control group (*p* = 0.3450). Additionally, a strong IgG response was also detected against the sand fly salivary protein LJL143 (Figure 1D). Interestingly, although all animals were immunized with the same dose of LJL143 (1 µg), a significant increase (*p* < 0.05) in reactivity against this protein was observed in the presence of the TLR-4 agonist adjuvant.

### 3.2. Immune Responses in Vaccinated Hamsters after L. infantum Infection

**Figure 2:**
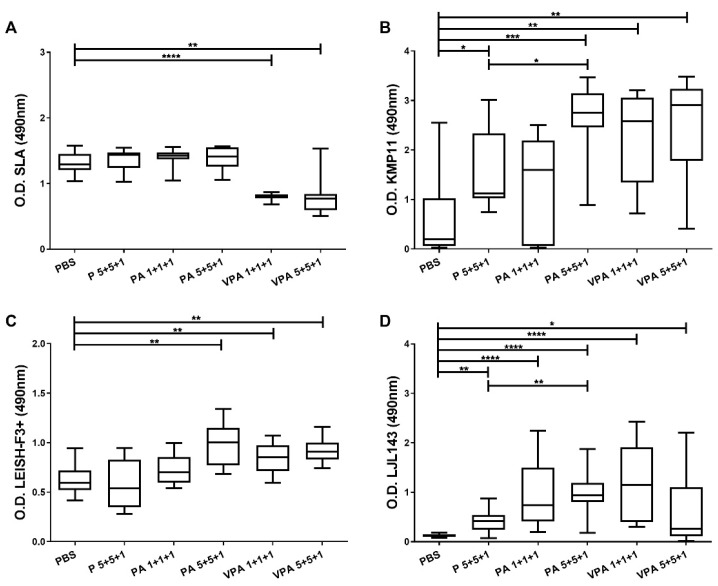
Specific IgG responses after *L. infantum* challenge. One month after the last immunization, the hamsters were infected intracardially with 2 × 10^7^ *L. infantum* stationary-phase promastigotes. Twelve weeks later, they were euthanized, and plasma was obtained. Specific antibody reactivity against SLA (**A**), and against the specific proteins KMP11 (**B**), LEISH-F3+ (**C**), and LJL143 (**D**), was assessed by ELISA. The results are shown as whisker (min to max) plots for each group (*n* = 9). Significant differences between groups are indicated (Mann–Whitney U test): * *p* < 0.05; ** *p* < 0.01; *** *p* < 0.001; **** *p* < 0.0001.

Four weeks after the last immunization, the hamsters were infected with 2 × 10^7^ *L. infantum* promastigotes, and 12 weeks later the humoral responses were examined. On one hand, a significant reduction was seen in SLA-specific IgG antibodies for the VPA 1+1+1 (*p* < 0.0001) and VPA 5+5+1 (*p* = 0.0055) groups compared to those in unvaccinated control hamsters (Figure 2A).

On the other hand, reactivity against the antigens included in the vaccine candidate was generally higher in the vaccinated groups versus the control group after infection. Differences were also observed depending on the antigen dose, the presence of the adjuvant, and the use of VS (Figure 2B–D). Reactivity against KMP11 increased significantly (*p* = 0.0133) in the animals vaccinated with 5 µg antigens together with the adjuvant (PA 5+5+1). However, no significant increase was observed for the PA 1+1+1 group compared to the control group (*p* = 0.4894), although the picture was different when the proteins were included in virosomes (VPA 1+1+1) (significant difference versus the control group, *p* = 0.0021). In fact, animals vaccinated with the complete vaccine formulation (VPA 1+1+1 and VPA 5+5+1), as well as those in the PA 5+5+1 group, showed the highest levels of anti-KMP11 antibodies (no significant differences among these three groups).

Similarly, with respect to LEISH-F3+, the presence of the adjuvant in the PA 5+5+1 group helped generate a stronger antibody response than that seen in the P 5+5+1 group. This response was greater than that recorded for the control group (*p* = 0.0028) and similar to that seen in the VPA 5+5+1 group (*p* = 0.0025). Of note, the antigen doses did not influence the reactivity when proteins were administered in VS (VPA 1+1+1 and VPA 5+5+1) (Figure 2C).

Importantly, sera from control animals did not react with the LJL143 protein (Figure 2D). In fact, the responses of the immunized and infected animals to this antigen were similar to those observed prior to infection (Figure 1D and Figure 2D). Additionally, as for the other antigens examined, the IgG response against LJL143 was stronger when the adjuvant was present (PA 5+5+1) (*p* = 0.0030).

**Figure 3:**
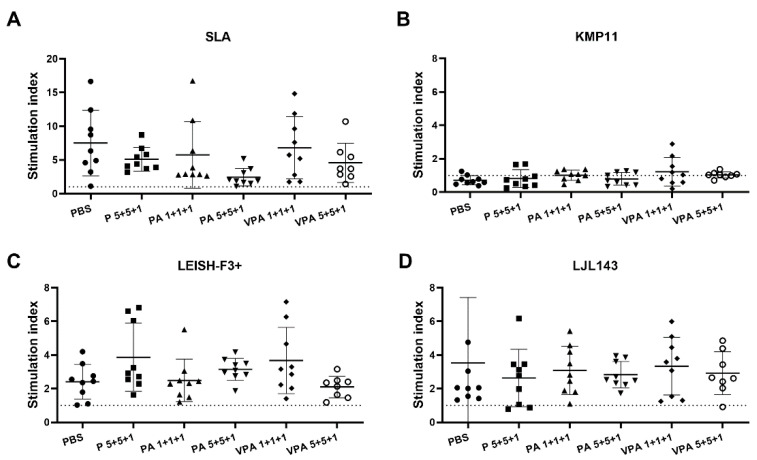
Antigen-specific lymphoproliferation assay in hamsters infected with *L. infantum*. Twelve weeks after infection, PBMCs were isolated from the peripheral blood of euthanized mice (*n* = 9 hamsters per group) and stimulated with SLA (**A**), KMP11 (**B**), LEISH-F3+ (**C**) or LJL143 (**D**) (10 µg/mL each) for 5 days at 37 °C. Lymphoproliferation was determined by the measurement of BrdU incorporation by ELISA. The results show the cell stimulation index—calculated as the ratio between the absorbance of antigen-stimulated cells and the result for non-stimulated cells (RPMI control)—of each animal in a scatter plot. Dotted lines represent the stimulation index obtained in the context of three naive hamsters.

The absence of cellular immunity against *Leishmania* is decisive in the progression of infection. Therefore, in line with our previous study [26], next, the proliferative capacity of hamster PBMCs was examined in response to SLA and the specific vaccine antigens KMP11, LEISH-F3+, or LJL143 (Figure 3). The proliferation of PBMCs in response to SLA was similar in all groups; in contrast to the humoral response, no significant differences due to vaccination were observed (Figure 3A). Interestingly, no proliferation was detected after KMP11 stimulation (Figure 3B). Differently, specific cellular responses to LEISH-F3+ and LJL143 were observed, but of a lower magnitude than that seen in response to SLA (Figure 3C,D). It is noteworthy that the specific lymphoproliferation against the vaccine antigens was no different from that seen in the control group. This is surprising in the case of LJL143 since the control animals had no contact with the antigen (Figure 3D). This finding indicates that proliferative PBMC responses against this protein require no immunization step.

### 3.3. The Complete Vaccine Formulation Confers Protection against L. infantum Infection in Hamsters

**Figure 4:**
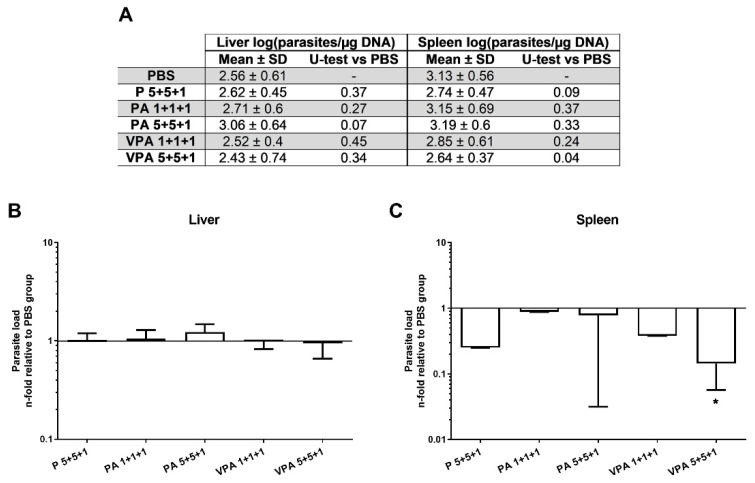
Parasite load in vaccinated hamsters after *L. infantum* infection. The parasite loads in the liver and spleen were analyzed by qPCR 12 weeks after infection (**A**). The results are represented as the mean ± SD of the relative units (RU) for each group (*n* = 9) in the liver (**B**) and spleen (**C**), calculated as the number of parasites per µg of hamster DNA with respect to the mean of the control group. Significant differences between vaccinated groups and the control group are indicated (Mann–Whitney U test): * *p* < 0.05.

To determine the efficacy of the proposed vaccine, the parasite load was measured by qPCR 12 weeks after infection in the target organs of VL. No differences were seen in the context of liver parasite burdens between immunized animals and controls (Figure 4A). Importantly, in the spleen, parasite load in the VPA 5+5+1 group was significantly lower—some 86% reduction—than in the control group (*p* = 0.0393) (Figure 4B).

**Figure 5:**
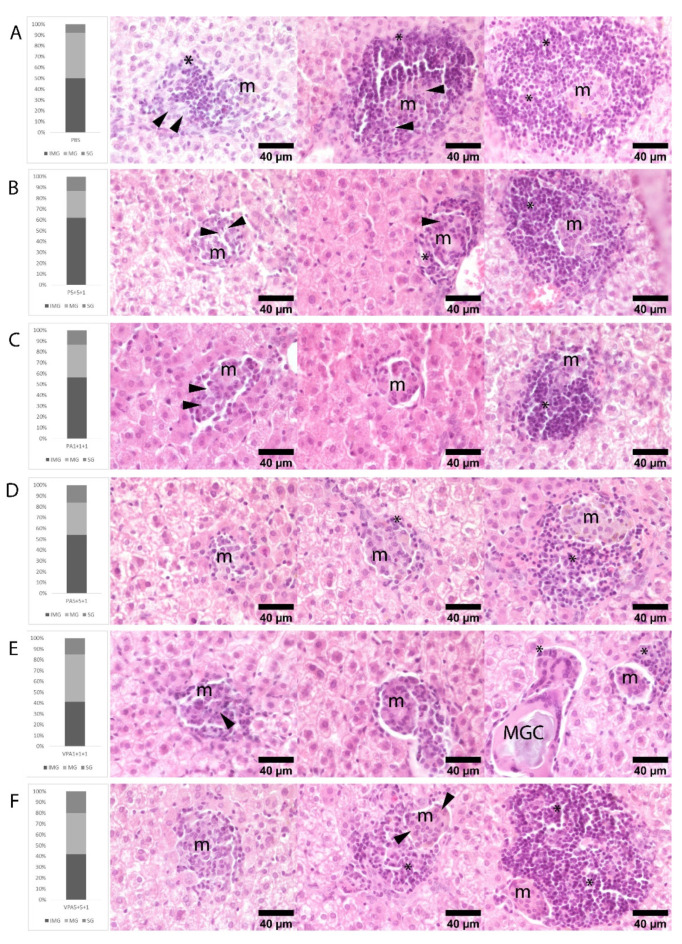
Histological findings in the liver of hamsters infected with *L. infantum.* Rows illustrate the percentage of immature (IMG), mature (MG), and sterile (SG) granulomas observed in hematoxylin-eosin stained liver sections of hamsters at 12 weeks post-infection: (**A**) PBS, (**B**) P 5+5+1, (**C**) PA 1+1+1, (**D**) PA 5+5+1, (**E**) VPA 1+1+1, and (**F**) VPA 5+5+1 groups. Immature granulomas consist of poorly defined aggregates of macrophages (m) and small numbers of intermixed lymphocytes (*). Mature granulomas are more organized and surrounded by small numbers of lymphocytes. Sterile granulomas contain small compact aggregates of macrophages surrounded by moderate to large numbers of lymphocytes, all devoid of amastigotes. Amastigotes in immature and mature granulomas are pointed out by arrows. Rare multinucleated giant cells (MGCs) containing membrane rests and mineral debris were seen mainly in SG and lesser in MG. *n* = 9 animals per group.

Protection was significant when the antigens were combined with adjuvants and presented in VS, as revealed by the granulomatous responses observed in the liver. The intracardiac inoculation of *L. infantum* promastigotes induced extensive granulomatous hepatitis. Three maturation stages of granulomas were observed in all six groups: immature, mature, and sterile granulomas. Amastigotes were found only in immature and mature structures. Statistical significance among the groups was not observed by comparing each of the stages of maturation; however, there was an overall predominance of immature and mature granulomas and the percentage of sterile granulomas was lowest in the control group and highest in the VPA 1+5+5 group. Groups PA 1+5+5 and VPA 1+1+1 also showed notable numbers of sterile granulomas (Figure 5). These results suggest that the vaccinated animals may show a greater capacity to form sterile granulomas, especially when the vaccine antigens were combined with adjuvants and vehiculated in VS.

## 4. Discussion

The present results show that this candidate vaccine elicits specific immune responses that are maintained after infection. However, a specific humoral response against KMP11 was sometimes not observed prior to infection, particularly in those animals that received the smaller dose of antigens. Moreover, as observed in the context of a mouse model, no IgG response was detected when KMP11 was included in VS [26]. Importantly, humoral responses seem to be influenced by the dose of KMP11 since, after infection, when the amount of KMP11 present is greater (due to exposure to *Leishmania* parasites), a specific humoral response against KMP11 was observed in all groups. It should be noted that the post-infection response against KMP11 was even stronger in animals administered with 5 µg of KMP11 and in those of both of the VPA groups. These results suggest that while the KMP11 dose in the PA 1+1+1, VPA 1+1+1, and VPA 5+5+1 groups might not be sufficient for the induction of humoral responses in the context of vaccination (at least under the present ELISA conditions), it is sufficient to elicit a significant increase in the anti-KMP11 response post-infection.

Similarly, increasing the dose of LEISH-F3+ led to a strong post-vaccination response. Of note, the humoral response to LEISH-F3+ post-vaccination was greater than that seen against KMP11 (all groups except the PA 1+1+1 group showed greater reactivity than the control group). Probably this stronger response against LEISH-F3+ is a consequence of combining the three antigens in one chimeric protein [18] (KPM11 is but a single antigen).

The present results also show that VS packaging enhances the humoral response against the LEISH-F3+ protein; the immune response to the 1 µg dose was equal to that of the 5 µg dose when VS were used (PA 1+1+1 < VPA 1+1+1 = VPA 1+5+5). This result is explained by the auto-adjuvant properties of VS [53]. Interestingly, no such observation was made in the earlier mouse study [26], perhaps due to differences between the model species.

An increase in the humoral response against the KMP11, LEISH-F3+, and LJL143 antigens was also induced by the GLA-SE adjuvant. Certainly, this adjuvant is known to enhance the IgG response to the immunogenic elements of the parasite, such as those included in the LEISH-F3+ protein [17]. Importantly, it also enhanced the responses to antigens from other organisms, as reported in the clinical trials of the PRIMVAC vaccine against malaria [54], the ID93 polyprotein vaccine against tuberculosis [30], the LepVax vaccine against leprosy [55], and an Ebola virus vaccine tested in mice [56]. Of note, the GLA-SE-mediated enhancement of the humoral response was associated with a Th1-type cellular response. For example, mice immunized with the KSAC polyprotein (formed by some of the *Leishmania* antigens included in the present study, (KMP11, SMT, and CPB)), when administered with GLA-SE, showed an increase in IFN-γ-producing T lymphocytes after infection [57]. Similarly, it has been shown that mice immunized with LEISH-F3+ and GLA-SE produced a specific Th1-type response against the parasite, based on IFN-γ, TNF, and IL-2 production [18]. The increase in the number of T CD4^+^ lymphocytes and their functionality has also been observed in humans administered with ID93 + GLA-SE [30]. Thus, the complete combination of factors for the present vaccine (i.e., proteins, VS, and GLA-SE) also has the potential to induce a specific Th1-type cellular response in hamsters. This was not specifically analyzed in the present study, but is probably the case, considering the results of previous work in a murine model [26].

A number of human, murine, and canine experimental VL models have established an association between the development and maturation of granulomas in the liver and spleen and resistance to infection [14,50,51,52]. Immature granulomas are ineffective at controlling *Leishmania* infection; they can, however, develop into more successful mature and sterile granulomas, which ultimately eradicate the parasites before decreasing in number, thus resolving the hepatic inflammation caused [51]. Importantly, the presence and number of sterile granulomas are strongly associated with the efficiency of the resolution of infection [50,51,52] and have been used to measure the effectiveness of VL vaccines in other animal models [14]. The percentage of mature and sterile granulomas in the present work may indicate the efficiency of the tested complete vaccine. Indeed, at 12 weeks post-infection, the group with the smallest percentage of sterile granulomas was the control group (eight percent sterile granulomas), while for the vaccinated groups this figure ranged from 13% to 20%. Although these percentages are low, the numbers indicate a certain degree of efficiency in infection resolution. Of note, the animals in the VPA 5+5+1 group showed the highest percentage of sterile granuloma formation, followed closely by those in the VPA 1+1+1 and PA 5+5+1 groups. These findings support the idea that reactivity against KMP11 and LEISH-F3+ is stronger when these antigens are administered in higher doses, within virosomes, and together with an adjuvant.

Our results also revealed a proliferative cellular response against the vaccine proteins—except for KMP11—in the animals that were vaccinated and later infected. This contrasts with the findings of our previous study, in which cellular responses specific to the KMP11 protein were detected in the spleen of immunized mice and in human PBMCs from asymptomatic and cured individuals [26]. A possible explanation for this discrepancy is that the cells of the infected hamsters may be in a state of anergy or exhaustion upon re-stimulation with this major *Leishmania* antigen [58]; additionally, the hypothesis that the hamster model has limitations in eliciting strong cellular responses after *Leishmania* infection may impair the detection of slight lymphoproliferative responses against KMP11. In addition, the nature of the antigen and the concentration used might have been critical for properly measuring KMP11 stimulation in T cells.

Additionally, the post-infection lymphoproliferative response seen against the vaccine proteins in the control animals was similar to that seen in the vaccinated animals. The latter similarity was also seen for the LJL143 protein. This is particularly interesting, since LJL143 is not a *Leishmania* protein, and the control animals were never exposed to *Lu. longipalpis* salivary antigens. In addition, the stimulation of human PMBCs from healthy donors with LJL143 is known to elicit a cellular response, although these were individuals from a *Phlebotomus perniciosus* endemic area and could have been exposed to sand fly salivary proteins [26]. Nevertheless, these results can be explained by the immunomodulatory properties of sand fly saliva, which is known to generate a strong immune response in naive cells in vitro [20]. This suggests that the use of salivary antigens in the context of anti-*Leishmania* vaccines may be advantageous [59]. In fact, preclinical results in mice showed that pre-immunization with LJL143 enhanced the lymphoproliferative response elicited against KMP11, LEISH-F3+, and SLA ex vivo [26].

Although no association was found between vaccination and enhanced lymphoproliferation in response to KMP11, LEISH-F3+, or LJL143 in the present animal model, the reduction in splenic parasite loads, and the increase in the number of sterile hepatic granulomas in those animals vaccinated with the complete formulation, both suggest that a protective cellular response does in fact occur. It is known that cellular exhaustion, histological changes, and the remodeling of the spleen during the progression of VL can limit—and even impede—antigen presentation, reducing the lymphoproliferative response against parasite antigens [60]. However, no loss of response against SLA was seen in the present work, probably because the progression of the infection in the control hamsters was insufficient to affect their lymphoproliferative capacity; indeed, the loss of cellular response after SLA restimulation is not observed until 17 weeks post-infection in hamsters [44], when the chronic and most severe phase of the disease is approaching (6 to 9 months) [18,61,62,63,64]. Nevertheless, the spleen is a secondary lymphoid organ, crucial to the development of systemic cellular responses against *Leishmania* parasites; therefore, the present results, indicating that the infection begins to be controlled in this organ, suggest that the immune response is more effective in vaccinated animals.

Interestingly, in animals immunized with the complete vaccine—which showed a smaller parasite load—significantly less IgG reactivity was seen against SLA (versus the control animals). Generally, the number of antibodies against *Leishmania* correlates with the severity of infection in the VL hamster model [65]. In fact, hypergammaglobulinemia is also one of the signs of VL in humans and is associated with parasite multiplication and the progression of infection [66,67]. This association is explained by the binding of antigen–antibody immune complexes (particularly of the Fc receptors) to macrophages and the consequent induction of IL-10 production together with the inhibition of IL-12, which tends to block Th-1 responses [67]. However, the influence of antibody responses in terms of pathological development depends on the maintenance of the splenic structure—which is required for adequate humoral response coordination [68]—and the quantity/type of antibodies produced [69]. It would be interesting to study the antibody subclasses present and the cytokines produced during the chronic phase of infection in the context of our vaccine candidate.

In conclusion, the present results show that this vaccine candidate, in its complete form and with the highest doses tested, is capable to elicit specific immune against *L. infantum* infection and reduce parasite load in hamsters. These results, together with others from studies involving common antigens or adjuvants, support the idea that toxicity studies should be undertaken, aiming at the future evaluation of vaccine safety in the context of phase I clinical trials in humans.

## Figures and Tables

**Table 1:** Experimental vaccination groups.

Group	Components
PBS	PBS 1
P 5+5+1	LEISH-F3 + (5 µg)/KMP11 (5 µg)/LJL143 (1 ug)
PA 1+1+1	LEISH-F3 + (1 µg)/KMP11 (1 µg)/LJL143 (1 µg)/GLA-SE (1 µg)
PA 5+5+1	LEISH-F3 + (5 µg)/KMP11 (5 µg)/LJL143 (1 µg)/GLA-SE (1 µg)
VPA 1+1+1	VS- LEISH-F3 + (1 µg)/VS-KMP11 (1 µg)/VS-LJL143 (1 µg)/GLA-SE (1 µg)
VPA 5+5+1	VS-LEISH-F3 + (5 µg)/VS-KMP11 (5 µg)/VS-LJL143 (1 µg)/GLA-SE (1 µg)

P: proteins; PA: proteins combined with the adjuvant; VS: virosomes containing KMP11, LEISH-F3+, or LJL143; VPA: VS including each protein individually, combined with the adjuvant.

## Data Availability

The data presented in this study are contained within the article.
